# Fostering teaching-learning through workplace based assessment in postgraduate chemical pathology residency program using virtual learning environment

**DOI:** 10.1186/s12909-020-02299-8

**Published:** 2020-10-23

**Authors:** Lena Jafri, Imran Siddiqui, Aysha Habib Khan, Muhammed Tariq, Muhammad Umer Naeem Effendi, Azra Naseem, Sibtain Ahmed, Farooq Ghani, Shahnila Alidina, Nadir Shah, Hafsa Majid

**Affiliations:** 1 Section of Chemical Pathology, Department of Pathology and Laboratory Medicine, Aga Khan University, Karachi Pakistan Stadium Road, Karachi, 74800 Pakistan; 2 Department of Medicine, Aga Khan University, Karachi, Pakistan; 3 Blended & Digital Learning Network, Aga Khan University, Karachi, Pakistan; 4 Department of Pathology and Laboratory Medicine, Aga Khan University, Karachi, Pakistan; 5 eLearning Developer, Department of I.T. Academics and Computing, Aga Khan University, Karachi, Pakistan

**Keywords:** Formative assessment, Feedback, Faculty training and engagement, Trainee development, Residency, Chemical pathology, Virtual learning environment

## Abstract

**Background:**

The principle of workplace based assessment (WBA) is to assess trainees at work with feedback integrated into the program simultaneously. A student driven WBA model was introduced and perception evaluation of this teaching method was done subsequently by taking feedback from the faculty as well as the postgraduate trainees (PGs) of a residency program.

**Methods:**

Descriptive multimethod study was conducted. A WBA program was designed for PGs in Chemical Pathology on Moodle and forms utilized were case-based discussion (CBD), direct observation of practical skills (DOPS) and evaluation of clinical events (ECE). Consented assessors and PGs were trained on WBA through a workshop. Pretest and posttest to assess PGs knowledge before and after WBA were conducted. Every time a WBA form was filled, perception of PGs and assessors towards WBA, time taken to conduct single WBA and feedback were recorded. Faculty and PGs qualitative feedback on perception of WBA was taken via interviews. WBA tools data and qualitative feedback were used to evaluate the acceptability and feasibility of the new tools.

**Results:**

Six eligible PGs and seventeen assessors participated in this study. A total of 79 CBDs (assessors *n* = 7 and PGs *n* = 6), 12 ECEs (assessors n = 6 and PGs *n* = 5), and 20 DOPS (assessors n = 6 and PGs n = 6) were documented. PGs average pretest score was 55.6%, which was improved to 96.4% in posttest; *p* value< 0.05. Scores of annual assessment before and after implementation of WBA also showed significant improvement, p value 0.039, Overall mean time taken to evaluate PG’s was 12.6 ± 9.9 min and feedback time 9.2 ± 7.4 min. Mean WBA process satisfaction of assessors and PGs on Likert scale of 1 to 10 was 8 ± 1 and 8.3 ± 0.8 respectively.

**Conclusion:**

Both assessors and fellows were satisfied with introduction and implementation of WBA. It gave the fellows opportunity to interact with assessors more often and learn from their rich experience. Gain in knowledge of PGs was identified from the statistically significant improvement in PGs’ assessment scores after WBA implementation.

## Background

Chemical pathology encompasses both practical laboratory and clinical skills. According to the guidelines of the College of Physicians and Surgeons of Pakistan (CPSP) 4 years’ training is required to satisfactorily complete the chemical pathology curriculum to the required depth and breadth. By the completion of training, the postgraduate (PG) trainees are expected to develop the desired competencies in the six major domains; medical expert, inter-professional communication, system based practice, professionalism, practice based learning and improvement and communication skills for independent practice. Interpretation and reporting is an important aspect of PG learning in chemical pathology [1–3]. This is accomplished mainly by discussing cases and laboratory investigations with peers and clinicians, examining preanalytical, analytical and post analytical problems before validating any biochemical result, investigating for the effect of interferences on test results and conducting regular clinical audits for quality and process improvements [4, 5]. Additionally that PG trainees of chemical pathology are closely involved with the consultants in performing procedures, provocative test and evaluating new tests for introduction in service [3]. A major part of a PG’s day is typically spent in clinical liaison: advising other clinicians about the appropriate laboratory tests for the investigation of a particular clinical problem, the interpretation of laboratory and clinical data and follow-up, and the effect of interferences on biochemical test results. Their curriculum is further complemented by structured case discussions and journal clubs usually held once a week in various institutions. While these teaching sessions focus on rare or unusual cases from old or new collections and are more structured; the teaching during such didactic teaching sessions often do not fulfill the purpose of critical thinking and reasoning [6]. A well facilitated journal club can become an important forum for teaching research methodology, clinical epidemiology and statistics, and seldom provides opportunities for training in clinical decision making and gaining critical appraisal skills [7–9]. There has been a concern PG trainees in chemical pathology are seldom observed, assessed, and given feedback during their work, while feedback is an integral teaching and learning tool. This has led to an increasing interest in a variety of workplace based assessment (WBA) methods that require observation and offer the opportunity for feedback in the clinical workplace [9, 10]. The principle of WBA is to assess trainees on work that they are actually doing and give feedback on their performance. The WBA has the benefit of high content validity through assessing actual performance of PG trainees in the workplace [10]. Benjamin Bloom et al. successfully classified students’ thought process into six very dynamic levels that increase in complexity, from knowledge as the baseline level, through comprehension, application, analysis, synthesis, to evaluation as the highest level [11, 12]. Frequent interactions of chemical pathology faculty with trainees working in high volume clinical laboratory can help identify students’ understanding, critical thinking ability in formulating a diagnosis, competency of laboratory procedures along with communication skills, attitude and decision making (reflected higher in Bloom’s taxonomy).

A virtual learning environment (VLE) is a distance learning platform for synchronous or asynchronous teaching that integrates course materials, assignments, assessments, and other tools [13–15]. For any healthcare institute a VLE can offer a place where students’ performance can be encouraged, managed, recorded and monitored [16, 17]. There is general paucity of literature on experiences of WBA implementation in subspecialties of pathology including chemical pathology. The appropriate use of WBA is still not yet studied and established in the chemical pathology community in our country and we felt the need to design and implement WBA program especially using a web-based model via VLE. As Coronavirus Disease (COVID-19) continues spreading in Pakistan and worldwide integrating VLE in postgraduate medical education has long term benefits. Using VLE in teaching and learning will give immediate educational benefits during this unprecedented crisis and will also build up the long term resilience of healthcare education systems. The goal of the current study was to gauge the feasibility and acceptability of a student driven WBA model in postgraduate residency program of chemical pathology using VLE and to determine the gain in knowledge via pre and posttest before and after the WBA implementation.

## Methods

### Research setting

A descriptive multi methods study was conducted in the section of chemical pathology, Department of Pathology and Laboratory Medicine, Aga Khan University (AKU), Karachi, Pakistan. The study was conducted from January to December 2019 and was introduced in the training process of chemical pathology residency program. The chemical pathology training program is a 5 year residency at Aga Khan University; accredited by CPSP. The program is a self-directed adult learning, systemically organized in the form of week by week structured bench rotations for learning analytical skills and rotations for enhancing their clinical skills in practice of biochemical laboratory medicine to diagnose disease and to manage patients. The PG trainees must acquire a detailed understanding of biochemical processes and the changes that occur in various diseases while rotating through the benches/ subsections of chemical pathology which include the following: routine chemistries and enzymes; therapeutic drug monitoring and toxicology, immunoassays, biochemical genetics laboratory, urolithiasis laboratory service, point of care testing, electrophoresis and protein laboratory. A major part of fellows’ time is spent in advising other clinicians about the appropriate tests for the investigation of a particular clinical problem, the interpretation of results and follow-up, performing procedures, provocative tests and evaluating new tests for introduction in service, assay optimization and validation and research.

### Eligibility criteria and ethical considerations

Before initiating the study the university’s ethical review committee’s permission was sought. All PGs registered at CPSP, including fellows of chemical pathology training program at AKU and fellows rotating in section of chemical pathology from other pathology specialties from January to December 2019 were eligible to participate in the study. Faculty, senior technologists and managers at the section of chemical pathology were taken as assessors. Seventeen assessors and six eligible PGs were contacted for written informed consent and all agreed.

### Data collection

To ensure that the outcomes achieved at the end of each phase meet their purpose, and that WBA program team members are properly prepared for the next phase we divided the WBA project into phases. To deliver the WBA program effectively the entire study process was divided into following seven phases:

#### Phase 1-development of assessment tools

The chemical pathology residency program has a different teaching learning and training environment which includes report interpretations and correlating with clinical data, clinical audits, clinical consultations, and laboratory procedures, provocative testing, quality assurance measures and complaint handling. Therefore it was essential to know that the selected WBAs tools were fit for purpose. Using Norcini AMEE guide, validated WBA tools were chosen and modified according to the needs of chemical pathology residency program by WBA project team [10]. These tools included; direct observation of practical skills (DOPS), evaluation of clinical events (ECE) and case based discussion (CBD). Multiple validated tools (CBD, DOPS and ECE) were to be used by assessors to assess knowledge, skills, professionalism and critical thinking of PGs. Literature on medical education states that multiple assessing formats provide a sound basis for assessment [18] Reliability of these WBA tools depend on how they are being utilized and also requires assessors training for making the best use of these tools [10]. Therefore all the tools were piloted (on two assessors and two PGs who were not included in the study) and reviewed (by five assessors who were part of the study). The WBA tools included items like case identification, PGs and assessors’ identification, knowledge/ skills/ attitude assessed, strengths and weaknesses of the PG, whether patient centered care and infection control advice was given. There were 28 items to be filled for DOPs, 26 for ECE and 23 for CBD on VLE. The WBA tools were structured such to include the competencies required by the PG trainees during their professional training which included medical expert, inter-professional communication, system based practice, professionalism, practice based learning and communication skills. The WBA tools were modified as per laboratory training requirements and items were made specific to the laboratory environment. Assessing components of every WBA tool were graded from 1 to 6 where 1–2 were marked ‘below expectations’, 3-‘borderline’, 4-‘meets expectations’ and 5–6 implied ‘above expectations’.

#### Phase II: outlining the WBA process

The process of WBA was clearly delineated with the goals of making it student driven and making it instrumental in the provision of feedback to PGs to navigate their learning towards desired outcomes. The PG was supposed to select a case (for CBD), any laboratory procedure (for DOPS) or complaint/ clinical audit (for ECE) which he/she has recently been involved with. The WBA process was to be initiated by PGs and they were supposed to select the assessor of their choice. For DOPS assessor was supposed to be present during the demonstration of the procedure by the PG. The discussion will start from and be centered on the PG’s record in the notes or reports and is designed to assess clinical decision-making and the application or use of medical knowledge in the care of patients. The discussion of assessors and PG would be followed by immediate feedback on assessment form uploaded on Moodle in another 5–10 min in the presence of the PG trainee. The assessments were to be performed against the standard expected at the end of the PG’s current stage of training and according to the level of complexity for each assessment. Guide for assessing complexity of cases, procedures or clinical events were prepared for the assessors. Low complexity WBAs were those that were uneventful and straight-forward with few demands made on the PG trainees, average complexity WBAs were those with manageable complications that most likely occur on a regular basis and high complexity WBAs were those that were difficult or unusual due to demanding encounters or unusual findings.

The primary goal of the assessment was to provide relevant comprehensive feedback to the PGs. Prior to submitting any of the three WBA tools PGs and assessors’ satisfaction level with the overall WBA process was recorded on VLE. Satisfaction level of both PGs and assessors was gauged separately on a Likert scale of 1–10 every time a WBA tool was filled. To maximize the educational impact of WBA, assessors were invited to provide qualitative comments for PGs on their overall performance in CBD, DOPS or ECE.

#### Phase III: utilizing innovative technology in WBA

For WBA program structure, activities, management and application Moodle (free of charge resource) was selected as the VLE platform. With support from the department of I.T. Academic and Computing, the WBA tools finalized in phase I of the study were developed on the VLE and all essential documents including WBA process and assessors’ guide were uploaded (Table 1). Description of all three WBA tools and guide to the whole process of conducting WBA was also provided on the VLE.

**Table 1:** Material developed for WBA on Moodle for assessors and PGs to access and utilize

Type of content	Description
**WBA Forms**	- Case based discussion (CBD)
	- Evaluation of clinical events (ECE)
	- Direct observation of practical skills (DOPS)
**Videos**	- Introduction to WBA program in Chemical Pathology
	- Workshop videos including role plays of CBD, ECE and DOPS
	- Real time CBD discussion and feedback video
**Documents**	- Process of WBA
	- List of Assessors
	- Residency Manual
	- Course Curriculum
	- Year wise Learning Objectives
	- Year wise Entrustable Professional Attributes
**Educational content**	- Review articles
	- Presentations
	- Book chapters

*Abbreviations*: *WBA* Workplace based assessment, *PGs* postgraduates, *CBD* Case-based discussion, *DOPS* Direct observation of practical skills, *ECE* evaluation of clinical events

#### Phase IV: introducing WBA program to the assessors

A half day hands-on WBA workshop to prepare the assessors to use the VLE for WBA was designed and executed face to face to 15 participants. Thorough hands on training through this workshop of all assessors was done with added objective of controlling rater bias. In-depth briefing regarding the entire process of WBA was given by the principal investigator (PI) and CO-PIs through various activities to determine the strengths, weaknesses, opportunities and threats (SWOT) of introducing WBA, along with hands-on use of WBA tools (CBD, DOPS and ECE) by role play. The SWOT analysis on WBA project was done in flipchart activity format with assessors by dividing them into four groups. Preliminary results from SWOT analysis were then detailed and the session invited comments and feedback using post-it’s to elaborate as described in Table 2. Positive attitude of assessors, documentation of informal teaching and awareness that this was the need of time were identified as major motivators. From the post workshop feedback the momentum, support and excitement of the workshop participants for the new process change could be appreciated.

**Table 2:** Findings of SWOT analysis of WBA project

**Internal**	**Strengths**	- WBA is conducive to the construction of a learning culture
		- Will identify PGs who need assistance
		- Will assist PGs in logbook entries
		- Documentation of faculty-PG interactions
	**Weaknesses**	- Faculty not comfortable with Moodle
		- Faulty time
		- WBA not integrated to current curriculum and assessment process
		- Student driven so student dependent
		- Low reliability
**External**	**Opportunities**	- Great demand for WBA
		- Great demand of using Moodle in the digital era
		- In line with institute’s goals
		- Support available from Digital and Teaching and learning network of the institute
		- If it works can be presented as a model at national level
	**Threats**	- Other institutes in the country may start WBA before us
		- May not work because of national policies

*Abbreviations*: *SWOT* strengths, weaknesses, opportunities, and threats, *PG* postgraduate, *WBA*: workplace based assessment

#### Phase IV: using training of trainers (TOT) approach

A full hands-on workshop was conducted by experts from department of I.T. Academic and Computing to prepare the assessors to use the VLE for WBA seven master trainers. The workshop was a complete training session with hands on practice of using Moodle on individual laptops. Following the TOT methodology the master trainers, given the title of ‘Moodle Champs’, then trained the rest of the assessors in the Section by small group discussions. They demonstrated how to use VLE for WBA in multiple small group discussions.

#### Phase V: training of the fellows

PG trainees were introduced to WBA with a presentation and eight to ten small group discussions on WBA and the use of VLE for teaching and learning. The whole WBA theory was explained and they were demonstrated the overall process of WBA. It was explained that it was for their benefit and to provide regular timely feedback to them. It was reiterated that in order to close the gap between actual performance and desired performance they need to actively take action and improve. Blueprint of the course curriculum was made available on VLE for them with the desired outcomes.

#### Phase VI: WBA program execution

Once WBA tools were developed, validated and tested on VLE, all assessors and PGs were trained and the process was clear to all participating the WBA process was implemented. Pretest of all PGs participating were conducted before WBA implementation and posttest was taken after 3 months of participating in WBA program. Being convenient, cost effective with the ability to cover broad content, multiple choice questions (MCQs) were chosen as the method of assessment for both pre and posttests. The MCQs based on CPSP chemical pathology residency program curriculum were developed by two content experts and uploaded on VLE. All 20 questions developed were scenario based and were structured for a higher cognition level of analyzing and interpretation. Data gathered during WBA execution were PGs performance through WBA tools, time spent in each WBA feedback and discussion and pretest and post test scores. Results of annual assessment before (year 2018) and after implementation (year 2019) of WBA were also evaluated to see the effect of WBA in PGs knowledge. Both type of assessments involved the same batch of PG trainees. The annual assessments of 2018 and 2019 were similar in content and structure using same table of specifications of chemical pathology course by paper setters. The assessments were blueprinted to the chemical pathology curriculum, ensuring that there was extensive assessment coverage of the curriculum content.

#### Phase VII: feedback and evaluation

Semi-structured individual interviews were conducted with randomly selected five assessors and PG trainees to gauge their response about the advantages of WBA, challenges faced and how they can be resolved. A quiet and convenient venue was chosen for the interviews. The interviews, which lasted 30 min to 1 h each, were conducted by one interviewer using a semi-structured interview format to promote a discussion and further questions. The interviewer, guided by the interview format, could direct questions in such a way as to facilitate more details and insight on the PG trainees’ and assessors’ responses. All questions were open ended. The main questions asked were as follows: how was their experience of using VLE for WBA and how could WBA program be improved? Data via interviews was being collected simultaneously from two sources; PG trainees and assessors. Data collection and analysis occur simultaneously so that the analyzed data guided subsequent data collection. The interviewer did not record the interviews but made written notes which were supplemented by extensions on those notes immediately following the end of the interview.

### Statistical analysis

#### Quantitative analysis

All data from recorded WBA on VLE were extracted into excel spreadsheets. Mean ± SD were generated for quantitative data (pre and posttests scores). Pre and posttests score comparison and comparison of results of annual assessment and after implementation of WBA were done using paired sample t test. *P* value < 0.05 was considered significant.

#### Qualitative analysis

For qualitative data frequencies were calculated. The feedback interviews were conducted and analyzed using the constructive grounded theory approach [18]. The notes from the interviews were read and modified for readability. Important comments relevant to the objective of the study were identified. The PI and research associate analyzed the feedback interviews to seek common concepts and opinions, grouping them into ‘codes’. Their independent results were compared and the identified phrases common to both investigators were used for further analysis. They met to debate their findings, confirming the codes and highlighting comments from the respondents that supported these. The individual codes were then sorted underneath 4 main themes from assessors’ interview and 3 from fellows through an iterative process of discussion and reflection.

## Results

Six eligible PGs and 17 assessors participated in this study. A total of 79 CBDs (assessors *n* = 7 and PGs *n* = 6), 12 ECEs (assessors n = 6 and PGs *n* = 5), and 20 DOPS (assessors n = 6 and PGs n = 6) were successfully recorded.

### Case-based discussions

The CBDs most frequently focused on clinical and laboratory data on an outpatient record (61/79, 77.2%) or inpatient record (18/79, 22.7%), and internal and external quality control summaries were also used. During the CBDs (*n* = 79), 7 assessing components were marked ‘borderline’ while 2 components were found to be ‘below expectations’ at various events. PGs were at par in most of the discussions. Suggestions for development were provided in 49.3% (*n* = 39) of CBDs (Table 3). Most (98.3%) of the CBDs were on data interpretation, some (25.7%) on lab management and few (8.6%) on professionalism. Majority of CBD (*n* = 64, 81.01%) recorded dealt with laboratory data interpretation followed by analytical (*n* = 21, 26.5%) and preanalytical (*n* = 15, 18.9%) issues. From the total cases in CBDs, 22.4% (*n* = 13) were of high complexity, 72.4% (*n* = 42) moderate and 5.2% (*n* = 3) of low complexity. Case mix included electrolyte imbalance, proteinuria, renal stones and renal failure, endocrinopathies (thyroid and parathyroid disorders, hyperprolactinemia, diabetes, growth disorders, insulinoma, hyperaldosteronism, Cushing Syndrome and Addison’s disease), autoimmune disorders, hypervitaminosis D, dyslipidemia, liver and pancreatic insufficiency, sepsis, malignancies and rare diseases (acute intermittent porphyria, maple syrup urine disease, lysine protein intolerance, and methylmalonic acidurias). The average time taken for CBDS’ discussion and feedback were 11.2 ± 5.3 min and 9.2 ± 7 min respectively. Out of the total CBDs conducted 41.7% (*n* = 33) got over within 20 min.

**Table 3:** Description of case complexity, satisfaction level of assessors and trainees and assessors’ feedback to PG trainees

		Assessment Tools		
		CBD	ECE	DOPS
	Frequency n (%)	79 (71.1)	12 (10.8)	20 (18)
**Case Complexity, n (%)**	**Low**	3 (3.8)	–	4 (19)
	**Medium**	62 (78.5)	9 (69.2)	15 (76.2)
	**High**	14 (17.7)	3 (30.7)	1 (4.7)
**Mean Satisfaction level on a scale of 1–10**	**Assessors Response (*n*** **= 17)**	8.23	8.15	7.77
	**Residents Response (n = 6)**	8.42	8.38	8.08
**Assessors Feedback to PG trainees (few narrative comments shown as examples)**	**Suggestions for Development**	• Revise the diagnostic criteria for multiple myeloma • Study Hartnups and Cystinuria • Difference between cobalamine defect and deficiency unclear. • How are internal and external quality controls practiced for pancreatic stool elastase testing? • Discuss another case with hypoparathyroid • Observe prolactin PEG precipitation procedure. • Study NCEP-ATP-III guidelines for dyslipidemia	• Read qQuality control chapter from Kaplan • Do some more literature review when handling complaints • Read carryover study • Study the peer group proficiency testing summary report • Delineate the process of complaint handling • Need to review the related reports prior to consultation • Study the type of laboratory errors	• Read standard operating procedures of growth hormone insulin procedure again • Will follow-up with you again on this complaint • More practice required • Read the sample receiving and processing policy • Improve understanding of chromatogram when performing kidney stone analysis • I will follow up with you as more practice is needed • Did not check specimen integrity and was not aware of the suboptimal specimens policy in the laboratory
	**Anything especially good**	• Knowledge of hypoglycemia cutoffs • Case selection was good, complete history of patient was available Knows the indication of urine amino acid testing • Collaborative approach of learning used • Understanding of chromatogram is good while reporting urine organic acid tests • All biochemical indices relevant to the case were correctly calculated and interpreted	• Good communication skills • Reliability in complaint solving • Followed all institutional processes correctly • Knows interpretation of method validation report • Can teach junior residents the interpretation of proficiency testing • Followed an emotional intelligence model for resolving queries	• Showed good confidence • Proper personal protective equipment was worn • Established good rapport with the patient • Good communication skills • Was able to demonstrate knowledge of the complications of procedure and corrective action • Effectively utilized cognitive skills systematically for problem solving during procedure

### Evaluation of clinical events

The ECEs were most frequently conducted patients with outpatient record (*n* = 12, 92.3%). Out of the total ECEs (n = 12) conducted, 50% (*n* = 6) were regarding consultation on laboratory data, 42% (*n* = 5) on complaint handling while no clinical audits were documented. All complaints or consultations discussed in ECE were of moderate complexity (*n* = 9, 69.23%) to high complexity (*n* = 4, 30.7%). Seven components of ECEs were marked ‘borderline’ at several times. Topics discussed in ECE were autoimmune disorders, anemia, malignancy, endocrinopathies, quality control and proficiency testing survey data and prenatal screening panel. Average time taken for discussion and feedback of ECEs were 8.7 ± 3.2 min and 7.7 ± 4.2 min respectively. Majority (66.6%, *n* = 8) of the time assessors and PGs got over with the discussion and feedback over within 20 min.

### Direct observation of procedural skills

Out of the 20 recorded DOPS, 70% (*n* = 14) were on biochemical analysis and instrument handling while 30% (*n* = 6) were on procedural skills. Six components of DOPS were marked ‘borderline’ several times. None of these WBA demonstrated evidence of ‘below expectation’ by the assessors. Instruments on which DOPS was demonstrated were ion selective electrode analyzer, point of care testing, centrifuges, Fourier transformed infrared spectroscopy and biochemical methods discussed were stone analysis, arterial blood gases analysis, and protein electrophoresis. Additionally there were DOPS carried out on safety measures to be taken when there is a biological and chemical spill. Majority (75%, *n* = 14) of the time assessors and PGs got over with the observation of DOPS, discussion and feedback within 20 min.

### Gain in knowledge

**Fig. 1:**
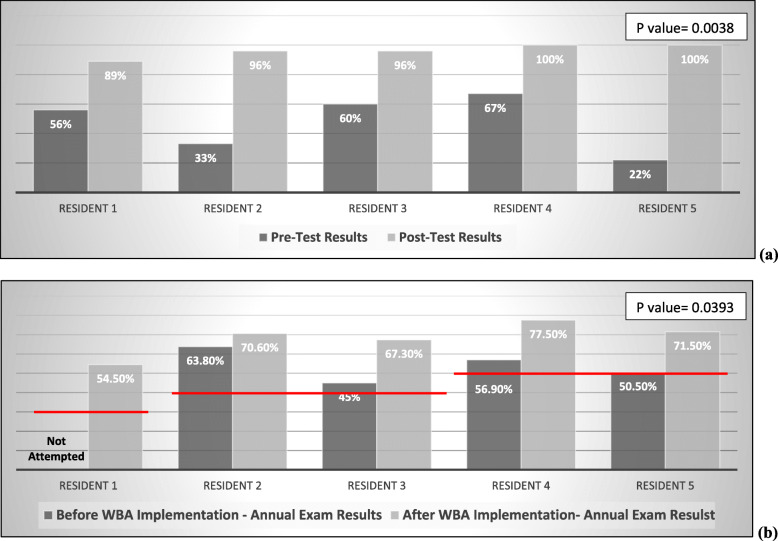
Residents’ (**a**) Pre and Post-Test Results and (**b**) Annual exam Results before and after implementation of the Workplace based Assessment using paired sample t test. The red lines represent the minimum required scores for the respective year of residency

The average pre and post scores were significantly different 55.6 and 96.4% respectively; *p* value 0.0038 (Fig. 1a). Results of annual assessment before (year 2018) and after implementation of WBA also showed significant difference, p value 0.039, shown in Fig. 1b.

### Acceptability and feasibility

Overall mean time taken to evaluate PGs was 12.5 ± 8.9 min and feedback time 8.9 ± 6.6 min. Mean WBA process satisfaction of assessors and PGs on Likert scale was 8 ± 1 and 8.3 ± 0.8 respectively. A total of five assessors were interviewed independently after informed consent. Four themes were derived from interviews after analysis by research associate and PI (Table 4). Challenges that the assessors faced, were mainly due to their first exposure to VLE as a teacher. One of the assessors mentioned that it was a two-way learning process. They always learned something new from the fellows while assessing them as they came well prepared. One of the assessor responded, *“The process of WBA has polished my assessment skills.* Fellows *are more up to date with recent developments which led to increase in my knowledge on various aspects as well”.* All of the assessors interviewed agreed that as per the format of WBA, the fellows were being assessed and provided feedback on regular basis and developed a feeling of accountability among fellows. An assessor stated *“I had been reading about WBA for a while but got a chance to implement it now, all senior technologists and lab manager were involved in assessment on daily basis which helped monitoring and keeping a close eye on the* fellows *daily activities”.* A senior assessor suggested *“Pilot should be presented at departmental level after successful completion and further taken to Postgraduate Medical Education for consideration to be incorporated into the system”.*

**Table 4:** Assessors’ and Postgraduate trainees’ feedback on introduction and execution of the WBA process in Postgraduate Chemical Pathology Residency Program

Themes Identified	Theme 1: Challenges that the faculty faced	Theme 2: How can WBA process be improved?	Advantages of WBA	
			Theme 3: To the Assessors	Theme 4: To PGs
**Assessors’ Responses**	Lack of interest on residents part and their laid back attitude	Residents need to show some rigor as its for their own development	Changed the format of informal teaching to formal	Assessed on daily basis making them accountable, focused & dedicated.
	A few Moodle related issue	Process should be made centrally operated through the department.	Two-way learning process. Always learned something new from the residents	Increased interaction with the faculty & opportunity to learn from their experience
	Forms can be further modified with emphasis on lab medicine	Give some weightage to the process and add in residents PGME evaluation	Polished assessment skills	Will come in handy to ace the exit exam
	Some difficulty in uploading teaching material on Moodle initially	Align WBA with Entrustable Professional Attributes	Efforts being documented	Whatever data is entered through WBA process can be used for logbooks entries
**Themes Identified**	**Theme 1: Challenges that the PGs faced**	**Theme 2: How can the WBA process be improved?**	**Theme 3: Advantages of WBA to PGs**	
**Residents’** **Responses**	The targeted number of CBDs that were needed was biggest challenge.	The number needs to reduce as its practically impossible to fulfill the required number	Opportunity to interact with the faculty more often than before and learn from them.	
	Faculty were a little strict with evaluation which led to unsuccessful attempts.	Our WBA process should align with ones happening in international institutes.	Increased exposure to bench work and instrument handling.	
	Should be given more access on Moodle to work on weaknesses highlighted by faculty.	WBA evaluation should be given some weightage and incorporated in PGME’s half-yearly/yearly’s assessment.	Identification of weak analytical skills.	
	Should be given enough access on Moodle to keep track of performance.	Moodle to be made more user friendly and encourage group discussions among residents when approaching a senior faculty for WBA.	Knowledge of lab safety enhanced.	

Five PGs were interviewed independently after informed consent. Three themes were derived from interviews after analysis by research associate and principal investigator (Table 4). All the PGs mentioned that it led to their development as they had the opportunity to interact with the assessors more often than before and learn from them. Besides, they had increased exposure to bench work and instrument handling and thorough study of topics they presented before the assessors. One of the fellows said *“I got to improve my bench skills, report interpretation, knowledge of lab safety, and identification of weak analytical skills”.* They all agreed that WBA was a great tool for learning, assessment and feedback. However, the number of target cases or bench mark should be realistic and suitable to PGs’ daily work load.

## Discussion

The fundamental rationale of this project was the provision of formative assessment and timely feedback to the trainees working in a busy high volume chemical pathology residency program. Formative assessment was introduced in the form of WBA in into the work environment of chemical pathology residency program. As with all methods of formative assessment, WBA methodology works best when they are embedded into the work environment, provide specific feedback, and are timely [19–22]. Current study has shown positive educational impact and good opportunities for feedback to PGs for all of the three formative WBAs investigated, judging by the feedback from both PGs and assessors.

Itin’s theory based on the Dewey’s design, proposed a ‘Diamond Model’ where the experiential learning process involves the educator and the learner in a transactive process. It takes into consideration the directional flow concept between educator, learner, learning environment and subject matter. In line with this theory, the current study also supports the approach to interpreting work based learning as an educational process which motivate learners to participate intellectually and physically in a rapidly changing work-related environment where they will go through the experiential process of creativity and innovation, with an aim to achieve successful outcomes. This has been included in the discussion with the addition of a new reference [23]. We report a high level of satisfaction among our assessors and PG trainees indicating that WBA can be successfully integrated into chemical pathology training program and day to day laboratory practice. A most important response in current study from PGs and assessors was that WBA conducted on real patients/ cases, in a clinical laboratory workplace by practicing pathologists, had true validity. All assessors felt that the PGs interaction with them had improved ever since the process was in place and stimulated a learning environment in the workplace. Whereas the PG trainees strongly felt that the feedback helped them ameliorate their performance, critical thinking and diagnostic ability. Most of the analysis was limited to quantitative data collected from the WBA forms but the qualitative feedback by the assessors to write narrative comments (Table 3) on the form gave PG trainees richer information and was highly appreciated by them than the numerical scores. This feedback from PG trainees is similar to feedback from trainees of other specialties and post graduate residency programs reported in literature [24–26]. All the PGs in their interview mentioned that this act of formative assessment and feedback led to their development in terms of exposure to bench work and instrument handling via DOPS and thorough in depth learning of topics presented during the CBDs. This substantiates the satisfaction of the process which gauged as 8 ± 1 and 8.3 ± 0.8 for the assessors and PGs respectively. Similar to the findings of the current study published literature on WBA suggests that it is a powerful means for changing the behavior of learners [27, 28]. Regarding average time spent by assessors to evaluate and provide feedback to PG’s was 12.6 min and 9.2 min respectively. Majority of the PGs got over with the discussion and feedback within 20 min. All the trainees were provided feedback right after the evaluation or assessment. As compared to WBA in other postgraduate training programs none of the trainees identified time pressures and difficulty in finding an assessor a hindrance for WBA [29, 30].

Workplace based assessments should be part of post graduate medical training programs with the correct selection of WBA tools [31]. A number of WBA tools or methods have been developed or regained prominence over the past couple of years Many WBA tools like mini-clinical evaluation exercise, ECE; CBD; DOPS, clinical work sampling, blinded patient encounters, and multi-source feedback have been extensively used and investigated by different educational experts across the globe [10, 32]. These WBA tools have been developed to provide a means of assessing clinical skills objectively, within the workplace, permitting assessment of the top layers of ‘Millers Pyramid’ or the ‘Pyramid of Competencies’ [33]. The top layer of the pyramid focuses on what happens in practice, and what the qualified professionals do in their workplace. The WBA tools selection for any post graduate medical education program should be as feasible, valid, and reliable measure of assessing PG trainee performance. Different types of learning require different methods of assessment [34]. The wide range and great depth of knowledge and the diversity of competencies to be incorporated throughout the training and their learning process means that multiple WBA tools might be required in the training programs. The selection of WBA tools in the current study was done in context of our daily workplace practices in the clinical laboratory. The purpose was that the assessment and feedback was integrated into their day to day work. The overall purpose of the three assessment methods that we used DOPS, ECE and CBD, was to provide structured timely feedback based on observed performance of PG trainees which we successfully achieved.

The advent of VLE and supporting software systems has made it possible to capture data for educational assessments in real time. We introduced VLE for execution of WBA program in phases. Conducting SWOT analysis in faculty development workshop helped in identifying lack of experience on VLE as the biggest weakness of the assessors. Faculty or assessors who have been brought up in a world with little or no technology can find it hard to use technology to enhance and support learning. One thing which is essential for sustaining this program on VLE is continuous administrative support, for facilitating and guiding faculty and trainees where needed.

Experiential learning in a WBA environment involves learners to follow a self-directed and proactive approach. Keeping this in view VLE ensures provision of continuum of stimuli where behavioral changes will take place through a range of responses from pleasant to non-aversive. The utility of VLE in this study concurs with the theory of Epstein S et al. Based on the contrasting views of the ‘cognitive unconscious’, which effectually deals with the learning experience and guides a person’s behavior using self-directed learning by the pupils [35]. The assessors were motivated to learn and recognized that VLE platform would be a potential route by which to document PGs feedback and formative assessment and overcome the challenge of lack of faculty time. Despite service pressured from the perceived positive educational impact in assessors’ interviews provides evidence of the practicability, acceptability and of this VLE-based formative assessment system. However it is important to understand that the design of learning strategies that are generally integrated in VLE are influenced by service pressures, which indirectly effects learning opportunities, leading to cognitive overload as reflected previously by Kilty et al. [36]. The cognitive overload, further limits a trainee’s time to reflect upon the case and discuss accordingly, ultimately effecting the learning curve.

The WBA program being implemented for the first time turned out to be a game changer in chemical pathology residency program at our institute. Successful implementation of WBA program was possible by engaging the team from the very beginning, making appropriate choices of WBA tools, advance planning, building of mutual trust, and training of assessors which also helped reduce rater bias and staying connected with PGs throughout the execution phase [37]. From our experience, we feel that continuous faculty development and administrative support are important factors that may influence the quality and sustainability of any WBA program in postgraduate medical education.

### Study limitations

One of the limitations of this study was that this was a single center study conducted on few PG trainees. The chemical pathology residency program is not huge with not many trainees across the country and even across the world [38]. The overall number of WBAs conducted by PGs may contribute in improving the validity of the study findings. A multicenter, multispecialty study design and larger sample size will improve its external validity. Another study limitation was that the standard setting for MCQs was not performed which questions the credibility of the assessment scores. The PG assessors (faculty, technologists and managers).employed in this study had different knowledge and expertise which could add to PGs’ assessment bias. To minimize this bias decision making cognitive skills of PG trainees were utilized to choose the assessor with expertise in the case or scenario to be discussed in WBA. Clinical cases and clinical complaints were brought up to faculty, procedures for DOPS to technologists/ faculty and administrative complaints to manager for assessment. Several other sources of error including distributional rater errors like leniency or severity errors (some assessors being more stringent and requiring a higher performance than other examiners who are more lenient) and range restriction error have not been addressed in the current study. However to control rater bias number of raters and number of encounters with PGs have been kept high. The dove and hawk analysis is one of many quality assurance steps that can be taken in the process of reducing the rater error in future such studies [39]. Despite the limitations, the current study has shown that it is feasible to implement WBA in chemical pathology residency program and PG trainees have derived educational benefits throughout this process. Multi center research using larger sample size of PG trainees and assessors from chemical pathology are required to validate our study findings.

## Conclusions

In postgraduate medical education trainings WBA is generally considered a component of assessment that evaluate how PG trainees in practice perform within their actual workplace. Supervision and feedback in daily clinical practice of PG trainees are of paramount importance in the work-based context of PGME. Many tools for WBA are available but it is imperative for the postgraduate medical education specialties to correctly choose and edit the WBA tools according to their clinical practice and needs. WBA has benefits for both assessors and fellows, therefore it is highly advocated for inclusion in assessment program for any competency based PG training. The high level of satisfaction from our respondents (assessors and PGs) indicates that WBA can be successfully integrated in a chemical pathology postgraduate training program. Further steps are to be taken to align WBA with the chemical pathology curriculum, EPAs and make the process acceptable at a national level. In short WBA implemented in chemical pathology had a catalytic effect creating more learning opportunities for the PG trainees and improving teaching and learning environment in the section.

## Supplementary information

**Additional file 1. Annexure 1 a**. WBA form for Case-based discussion (CBD). **b**. WBA form for Direct observation of practical skills (DOPS). **c**. WBA form for Evaluation of clinical events (ECE).

## Data Availability

The datasets used and/or analyzed during the current study are available from the corresponding author on reasonable request.

## Abbreviations

WBA: Workplace based assessment
PGs: Postgraduates
CBD: Case-based discussion
DOPS: Direct observation of practical skills
ECE: Evaluation of clinical events
VLE: Virtual learning environment
AKUH: Aga Khan University and Hospital
CPSP: College of Physicians and Surgeons of Pakistan
TOT: Training of trainers
MCQs: Multiple choice questions
