# Supplementary material for: Fostering teaching-learning through workplace based assessment in postgraduate chemical pathology residency program using virtual learning environment
Source: BMC Med Educ. 2020 Oct 23;20:383. doi: 10.1186/s12909-020-02299-8 (PMC7582426; doi:10.1186/s12909-020-02299-8)
Supplement: Supplementary file 1 — Additional file 1. Annexure 1 a. WBA form for Case-based discussion (CBD). b. WBA form for Direct observation of practical skills (DOPS). c. WBA form for Evaluation of clinical events (ECE). [file 12909_2020_2299_MOESM1_ESM.zip › Annexure 1aR1.doc]

**Annexure 1a: Case-based discussion (CBD)**

**Postgraduate trainee’s name:__________________**

**Clinical setting:	Outpatient	Inpatients**

**Patient ID:___________**

**Disorder or Condition being discussed: ________________**

**Clinical Problem category: Preanalytical	Analytical	Post analytical**

**Focus of clinical encounter: Data interpretation	Management	Professionalism**

**Case complexity: Low	Medium	High**

| **Please grade the following areas using the scale below:** | 1  Below expectations | | 2  Below expectations | | 3  Borderline | | | 4  Meets expectations | 5  Above expectations | 6  Above expectations | | U/C unable to comment |
| --- | --- | --- | --- | --- | --- | --- | --- | --- | --- | --- | --- | --- |
| Was the PG trainee able to explain pathophysiology of the disorder? |  | |  | |  | | |  |  |  | |  |
| Was the PG trainee able to identify/ suggest additional investigations needed in the case? |  | |  | |  | | |  |  |  | |  |
| Was the PG trainee able to deliver or suggest further clinical management? |  | |  | |  | | |  |  |  | |  |
| Was the PG trainee able to address patient/laboratory safety related issues pertinent to the case? |  | |  | |  | | |  |  |  | |  |
| Does the PG trainee know how to follow up this case? |  | |  | |  | | |  |  |  | |  |
| How was the PG trainee’s overall laboratory & clinical judgment? |  | |  | |  | | |  |  |  | |  |
| Overall professionalism |  | |  | |  | | |  |  |  | |  |
| Anything especially good? |  | | | | | | | | | | | |
| Suggestions for development |  | | | | | | | | | | | |
| Agreed action |  | | | | | | | | | | | |
| PG satisfaction with CBD | 1  Not at all | 2 | | 3 | 4 | | 5 | 6 | 7 | 8 | 9 | 10  Highly |
| Faculty satisfaction with CBD | 1  Not at all | 2 | | 3 | 4 | | 5 | 6 | 7 | 8 | 9 | 10  Highly |
| Time taken for feedback in minutes | | | | | | Time taken for discussion in minutes | | | | | | |
| Assessor’s name | | | | | | Date | | | | | | |
