# Supplementary material for: Fostering teaching-learning through workplace based assessment in postgraduate chemical pathology residency program using virtual learning environment
Source: BMC Med Educ. 2020 Oct 23;20:383. doi: 10.1186/s12909-020-02299-8 (PMC7582426; doi:10.1186/s12909-020-02299-8)
Supplement: Supplementary file 1 — Additional file 1. Annexure 1 a. WBA form for Case-based discussion (CBD). b. WBA form for Direct observation of practical skills (DOPS). c. WBA form for Evaluation of clinical events (ECE). [file 12909_2020_2299_MOESM1_ESM.zip › Annexure 1bR1.doc]

**Annexure 1b: Direct observation of practical skills (DOPS)**

**Postgraduate trainee’s name:__________________**

**Procedure/ Instrument name:__________	Patient ID:___________**

**Clinical Problem category: Procedure	Instrument Handling**

**Number of times procedure performed or instrument handled by PG: 0	1-4	5-9	>10**

**Procedure complexity:	Low	Medium	High**

| **Please grade the following areas using the scale below:** | 1  Below expectations | | 2  Below expectations | | 3  Borderline | | | | 4  Meets expectations | | 5  Above expectations | | | 6  Above expectations | | U/C unable to comment | |
| --- | --- | --- | --- | --- | --- | --- | --- | --- | --- | --- | --- | --- | --- | --- | --- | --- | --- |
| Did the PG trainee demonstrate understanding of indications for and scientific principles of procedure?/instrument? |  | |  | |  | | | |  | |  | | |  | |  | |
| Did the PG trainee obtain informed consent from patient/ guardian before starting procedure? |  | |  | |  | | | |  | |  | | |  | |  | |
| Did the PG trainee display consideration of health and safety requirements? |  | |  | |  | | | |  | |  | | |  | |  | |
| Does the PG show familiarity with and understanding of Standard Operating Procedures? |  | |  | |  | | | |  | |  | | |  | |  | |
| Did the PG trainee demonstrate technical ability & correct use of instrument? |  | |  | |  | | | |  | |  | | |  | |  | |
| Did the PG trainee ensure patient and colleague safety? |  | |  | |  | | | |  | |  | | |  | |  | |
| Did the PG trainee demonstrate consideration of quality control and assurance? |  | |  | |  | | | |  | |  | | |  | |  | |
| Was the PG trainee able to elaborate post procedure management? |  | |  | |  | | | |  | |  | | |  | |  | |
| Was the PG trainee able to seek help where appropriate? |  | |  | |  | | | |  | |  | | |  | |  | |
| Did the PG trainee demonstrate effective communication skills? |  | |  | |  | | | |  | |  | | |  | |  | |
| How was the PG trainee's overall ability to perform procedure or handle instrument? |  | |  | |  | | | |  | |  | | |  | |  | |
| Was the PG trainee able to display professionalism? (patient confidentiality, respect) |  | |  | |  | | | |  | |  | | |  | |  | |
| Anything especially good? |  | | | | | | | | | | | | | | | | |
| Suggestions for development |  | | | | | | | | | | | | | | | | |
| Agreed action |  | | | | | | | | | | | | | | | | |
| PG satisfaction with DOPS: | 1  Not at all | 2 | | 3 | | 4 | | 5 | | 6 | | 7 | 8 | | 9 | | 10  Highly |
| Assessor’s satisfaction with DOPS | 1  Not at all | 2 | | 3 | | 4 | | 5 | | 6 | | 7 | 8 | | 9 | | 10  Highly |
| Time taken for feedback in minutes | | | | | | | Time taken for discussion in minutes | | | | | | | | | | |
| Assessor’s name | | | | | | | Date | | | | | | | | | | |
