# Supplementary material for: Fostering teaching-learning through workplace based assessment in postgraduate chemical pathology residency program using virtual learning environment
Source: BMC Med Educ. 2020 Oct 23;20:383. doi: 10.1186/s12909-020-02299-8 (PMC7582426; doi:10.1186/s12909-020-02299-8)
Supplement: Supplementary file 1 — Additional file 1. Annexure 1 a. WBA form for Case-based discussion (CBD). b. WBA form for Direct observation of practical skills (DOPS). c. WBA form for Evaluation of clinical events (ECE). [file 12909_2020_2299_MOESM1_ESM.zip › Annexure 1cR1.doc]

**Annexure 1c: Evaluation of clinical events (ECE)**

**Postgraduate trainee’s name:__________________**

**Clinical setting:	Outpatient	Inpatients**

**Patient ID:___________**

**Complaint/ Disorder/ Patient encounter being discussed: ________________**

**Clinical Problem category: Preanalytical	Analytical	Post analytical**

**Focus of clinical encounter: Complaint	Query	Consultation /Report interpretation**

**Case complexity: Low	Medium	High**

| **Please grade the following areas using the scale below:** | 1  Below expectations | | 2  Below expectations | | 3  Borderline | | | 4  Meets expectations | | 5  Above expectations | | | 6  Above expectations | | U/C unable to comment | |
| --- | --- | --- | --- | --- | --- | --- | --- | --- | --- | --- | --- | --- | --- | --- | --- | --- |
| Does the PG trainee understand the theory of encounter/event (process)? |  | |  | |  | | |  | |  | | |  | |  | |
| Was the PG trainee able to apply clinical/pathological knowledge appropriately? |  | |  | |  | | |  | |  | | |  | |  | |
| Did the PG trainee make appropriate clinical judgements? |  | |  | |  | | |  | |  | | |  | |  | |
| Did the PG trainee follow established procedure? ( approved procedure or guidelines) |  | |  | |  | | |  | |  | | |  | |  | |
| Was the PG trainee able to demonstrate appropriate communication skills? (verbal and written) |  | |  | |  | | |  | |  | | |  | |  | |
| Did the PG trainee maintain patient focus and deliver patient centered care? (e.g. respect for patient dignity, consent, confidentiality, turnaround times) |  | |  | |  | | |  | |  | | |  | |  | |
| Was the PG trainee able to maintain professional standards? |  | |  | |  | | |  | |  | | |  | |  | |
| Does the PG trainee consider professional issues like record keeping, consultation with colleagues, linkage of department to others, plan for feedback? |  | |  | |  | | |  | |  | | |  | |  | |
| Was the PG trainee able to display organization & efficiency? |  | |  | |  | | |  | |  | | |  | |  | |
| Overall clinical care |  | |  | |  | | |  | |  | | |  | |  | |
| Anything especially good? |  | | | | | | | | | | | | | | | |
| Suggestions for development |  | | | | | | | | | | | | | | | |
| Agreed action |  | | | | | | | | | | | | | | | |
| PG trainee satisfaction with ECE | 1  Not at all | 2 | | 3 | 4 | 5 | | | 6 | | 7 | 8 | | 9 | | 10  Highly |
| Faculty satisfaction with ECE | 1  Not at all | 2 | | 3 | 4 | 5 | | | 6 | | 7 | 8 | | 9 | | 10  Highly |
| Time taken for feedback in minutes | | | | | | | Time taken for discussion in minutes | | | | | | | | | |
| Assessor’s name | | | | | | | Date | | | | | | | | | |
